# Arcanobacterium Brain Abscesses, Subdural Empyema, and Bacteremia Complicating Epstein-Barr Virus Mononucleosis

**Published:** 2018-02-28

**Authors:** Victoria Poplin, David S. McKinsey

**Affiliations:** 1 University of Kansas Medical Center, Department of Internal Medicine, Kansas City, KS; 2 Infectious Disease Associates of Kansas City, Kansas City, MO

**Keywords:** Arcanobacterium, Epstein Barr virus infections, brain abscess, steroids, infectious mononucleosis

## Introduction

Infectious mononucleosis is common among adolescents and young adults.^1^ Although most cases resolve spontaneously, several life-threatening manifestations have been recognized. No guidelines for management of mononucleosis have been published. However, the American Academy of Pediatrics recommends that a short course of corticosteroid therapy may be considered for certain serious complications, such as impending airway obstruction, massive splenomegaly, hemolytic anemia, hemophagocytic lymphohistiocytosis, or myocarditis.^2^ Although some physicians prescribe prednisone for symptomatic treatment of other manifestations of mononucleosis such as sore throat without airway obstruction, a recent Cochrane reviewidentified no apparent benefit of steroid therapy for uncomplicated mononucleosis^3^; such treatment is not recommended despite its widespread use.

Sinusitis is a well-recognized complication of viral infections, including mononucleosis. Corticosteroid therapy, even when only prescribed for brief periods, increases the risk of a variety of bacterial, viral, mycobacterial, and parasitic infections by 60%.^4^ Fardet et al. reported a 5.84-fold increased relative hazard of lower respiratory tract infections among patients who received systemic steroid treatment for any reason (the prevalence of upper respiratory tract infections including sinusitis was not studied) 5.

*Arcanobacterium haemolyticum* is a slowly growing, facultative anaerobic gram-positive bacillus.^6^ Originally named *Corynebacterium haemolyticum* when recognized as a cause of exudative pharyngitis in US soldiers and natives of the South Pacific in 1946, the organism’s name was changed to *Arcanobacterium haemolyticum* in 1982 after cell wall components and chemotaxonomic features were noted to differ from Corynebacterium *spp*. Humans are the primary reservoir for *A. haemolyticum,* which has two biotypes, rough and smooth. The smooth biotype is a cause of pharyngitis in healthy adolescents whereas the rough biotype is associated with a variety of invasive infections which are more commonly seen in immunocompromised adults.^6^ The aptly-named Arcanobacterium (“secret bacterium”) is difficult to cultivate with conventional culture techniques (i.e., incubation of specimens on sheep’s blood agar for 24 hours). One study reported that yield was highest when horse blood agar was utilized and specimens were incubated with supplemental 5% CO_2_ incubation for 48 hours.^7^ However, community laboratories do not typically use horse blood agar; thus the true prevalence of Arcanobacterium infections is likely higher than has been reported.

We report a case of Epstein-Barr Virus (EBV) mononucleosis treated with prednisone complicated by secondary bacterial sinusitis with intracranial extension leading to brain abscesses and subdural empyema, associated with Arcanobacterium bacteremia. To our knowledge this is the first report of EBV-associated *Arcanobacterium haemolyticum* brain abscess and subdural empyema.

## Case Report

A previously healthy 20-year-old African American male college football player developed sore throat, fatigue, and painful cervical lymphadenopathy. The diagnosis of infectious mononucleosis was established on the basis of a positive heterophile antibody test. One week later he had worsening sore throat and headache. Prednisone was prescribed for 10 days. Over the following two weeks he had recurrent fevers, chills, malaise, anorexia, and insomnia, but delayed seeking further medical attention because he was busy taking final examinations. His symptoms progressively worsened.

Three days prior to hospitalization he developed global headache, copious green rhinorrhea, recurrent rigors, and severe anorexia. He presented at a hospital emergency department where he appeared acutely ill. Posterior cervical lymph nodes were swollen and tender. No pharyngeal erythema or exudate was noted. No focal deficits were identified on neurologic examination.

His white blood cell count (WBC) was 16,300/μL (64% neutrophils, 24% bands, 7% monocytes, 5% lymphocytes). Total bilirubin was 5.7 U/L (direct 4.0 U/L), AST 410 U/L, ALT 290 U/L, and alkaline phosphatase 331 U/L. Epstein Barr virus viral capsid IgM serology was positive; viral capsid IgG serology was negative.

Computerized tomography scan of the head without contrast showed pansinusitis without intracranial masses, edema, or midline shift. Cerebrospinal fluid WBC count was 2,800/μL (46% neutrophils, 44% lymphocytes), glucose 44 mg/dL, and protein 100 mg/dL. Gram’s stain showed no organisms; culture, which was submitted prior to initiation of antibiotic therapy, was negative. Cerebrospinal fluid EBV PCR was positive (not quantified); 16s RNA was negative.

**Figure 1:**
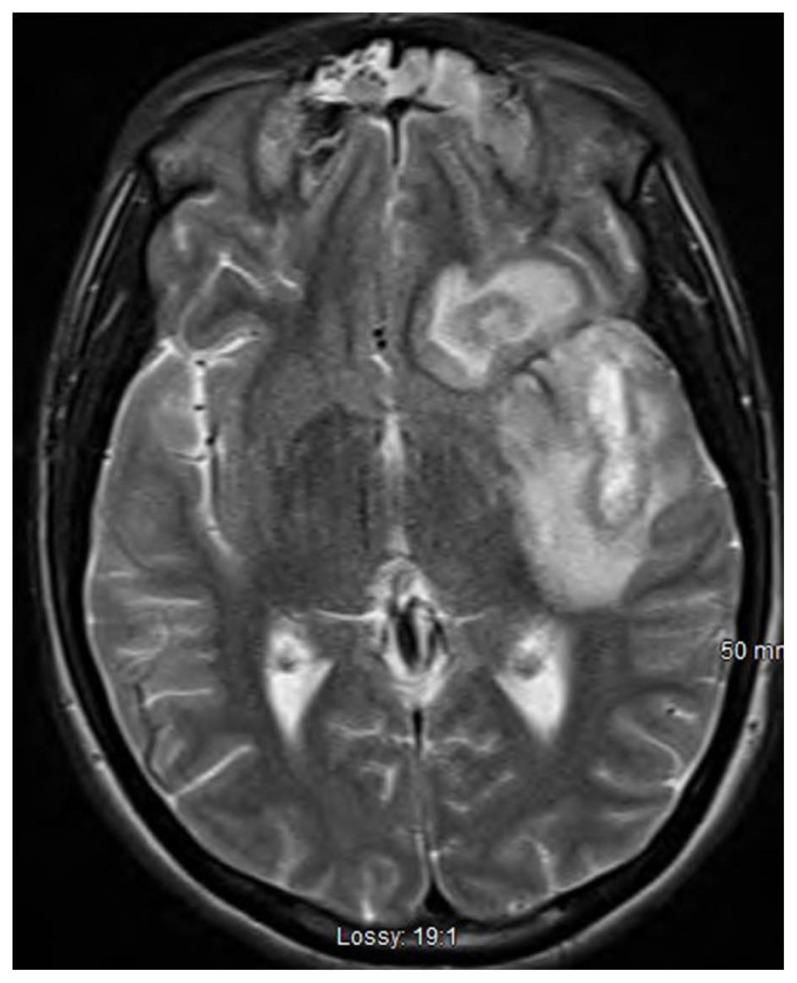
MRI scan of the brain, with gadolinium, demonstrated multilocular fluid collections with enhancing margins in the left temporal lobe and posterior inferior left frontal lobe, and a subdural fluid collection in the anterior and inferior left cranial fossa.

The patient was hospitalized and treated with ceftriaxone, doxycycline, and vancomycin. Doxycycline was discontinued after four days. Two of two sets of blood cultures grew *Arcanobacterium haemolyticum*; identification of the organism was confirmed by Matrix-assisted laser desorption/ionization (MALDI TOF) testing. Over the following four days the patient complained of worsening headache. MRI scan of the brain, with gadolinium, on hospital day five demonstrated multilocular fluid collections with enhancing margins in the left temporal lobe and posterior inferior left frontal lobe, consistent with abscesses, and a subdural fluid collection in the anterior and inferior left cranial fossa (Figure 1). Metronidazole was added to his treatment regimen.

The patient underwent image-guided pterional craniotomy with evacuation of frontal and temporal brain abscesses and removal of subdural empyema. Purulent fluid was encountered. Gram’s stain showed few polymorphonuclear leukocytes and rare gram positive bacilli with morphology characteristic of Arcanobacterium; culture was negative. He also underwent bilateral maxillary antrostomy, total ethmoidectomy, frontal sinusotomy, sphenoidotomy, sinus irrigation, and submucosal reduction of the inferior turbinates. Thick mucopurulent fluid was encountered. Culture was negative.

Following evacuation of the brain abscesses and subdural empyema, the patient’s clinical status improved substantially. His fever and headaches abated. He received a nine-week course of intravenous ceftriaxone and metronidazole. Repeat imaging at the completion of his treatment demonstrated resolution of intracranial abscesses. His infection has not recurred during a two-year follow-up period.

## Discussion

We report a case of EBV mononucleosis treated with prednisone complicated by secondary bacterial sinusitis with intracranial extension leading to brain abscesses and subdural empyema, associated with Arcanobacterium bacteremia. The dominant causative organism of the brain abscess and subdural empyema likely was Arcanobacterium: although abscess culture was negative following several days of antibiotic therapy, abscess fluid Gram’s stain showed only gram positive bacilli with an appearance consistent with Arcanobacterium. We postulate that EBV infection caused disruption of upper respiratory tract epithelium, triggering secondary bacterial sinusitis with subsequent contiguous spread of infection to the brain and subdural space in the setting of transient immunosuppression precipitated by EBV and exacerbated by prednisone therapy. To our knowledge, this is the first report of EBV-associated *Arcanobacterium haemolyticum* brain abscess and subdural empyema.

Pharyngitis is the most commonly recognized form of* A. haemolyticum* infection.^8^ In half of cases a prominent erythematous rash is seen. Arcanobacterium accounts for 2% of cases of pharyngitis in 15 – 18 year old healthy males. Several other forms of Arcanobacterium infection have been reported, including sinusitis^9^, pneumonia^10^, bacteremia^11^, meningitis^12^, and brain abscess.^10^,^13^–^15^ Bacteremia is rare. Two cases were identified during a 15-year period at the Karolinska Institute.^16^ A similar experience was reported at Ben Taub Hospital in Houston, where among 280,000 blood cultures from 2000 – 2015, only two grew Arcanobacterium.^13^ Skin and soft tissue infections typically are seen in older men who either are immunocompromised or have an underlying disease such as diabetes mellitus.^10^, ^16^

The association of life-threatening Arcanobacterium infection with a concomitant Epstein Barr virus infection was of interest in our case. In invasive infections such as brain abscess, bacteremia, pneumonia, and Lemierre’s syndrome, *A. haemolyticum* typically acts as a co-pathogen with other bacteria or viruses, for reasons that remain unclear.^11^,^14^,^17^ Monomicrobial invasive Arcanobacterium infections, in contrast, are uncommon. In a retrospective study, all six patients with Arcanobacterium bacteremia were found to have co-infection with a second bacterial species.^18^ The second bacterium presumably acted synergistically with *A. haemolyticum* to enhance its pathogenicity.

Although both Epstein Barr Virus mononucleosis and *A. haemolyticum* pharyngitis are encountered most commonly in persons between the ages of 15 – 24, concomitant infection has been recognized infrequently.^9^,^14^,^19^–^21^ Three cases of *A. haemolyticum* bacteremia with primary EBV infection have been reported, one of which had secondary bacterial sinusitis and polymicrobial bacteremia.^20^ However, this association may be more common than has been widely recognized. A study of 13 patients with *A. haemolyticum* bacteremia detected heterophile antibody positivity in five cases.^9^ Secondary bacterial infections (most commonly caused by beta hemolytic streptococci) are known to be associated with EBV, likely due to either temporary immune suppression or co-pathogenicity.^22^,^23^ Thus, transient humoral and cell mediated immunosuppression induced by EBV may enhance *A. haemolyticum’s* virulence.^9^,^14^,^21^,^23^,^24^ Steroid-induced immunosuppression would be expected to exacerbate the severity of EBV-Arcanobacterium coinfection.

Brain abscess can be caused by bacteria, mycobacteria, fungi, or parasites, and most commonly arises from either contiguous spread of infection, hematogenous seeding, penetrating trauma, or neurosurgical procedures.^25^ A variety of bacteria can cause brain abscesses; the implicated organisms vary based on the initial source of infection. Brain abscesses secondary to sinusitis, as in this case, usually are caused by anaerobic and aerobic streptococci, Bacteroides*, E. coli*, Enterobacter, Klebsiella, Proteus, S. aureus or Haemophilus.^23^ However in 13 – 25% of cases, brain abscess cultures are negative, likely secondary to antecedent antibiotic exposure or infection with non-cultivable microorganisms.^23^,^25^ *Arcanobacterium haemolyticum* is a rare cause of brain abscess; only six cases have been reported previously. Two of these cases were presumed odontogenic infections (one occurred post-dental extraction; a second patient had severe dental caries;^17^ a third occurred after penetrating skull trauma in a patient with acute sinusitis.^15^ The sources of the other three infections were not identified.^14^,^24^,^25^

The optimal treatment regimen for invasive *A. haemolyticum* infections has not been determined. Susceptibility testing is not standardized. *In vitro* data suggest that *A. haemolyticum* is susceptible to most classes of antibiotics, including penicillins, cephalosporins, clindamycin, carbapenems, macrolides, fluoroquinolones, tetracyclines, rifampin, and vancomycin, and resistant to sulfonamides.^26^ First line therapy with either penicillin or a cephalosporin is recommended. In β-lactam allergic patients, alternative options include clindamycin, doxycycline, fluoroquinolones, or vancomycin. Our patient had an excellent response to ceftriaxone therapy.

## Conclusion

*Arcanobacterium hemolyticum* bacteremia, brain abscesses, and subdural empyema developed in a young man with concomitant EBV mononucleosis complicated by sinusitis who received prednisone therapy. The association between EBV and Arcanobacterium may be more common than previously recognized, as Arcanobacterium is a slowly growing organism that does not propagate well on commonly utilized sheep’s blood agar. Treatment with prednisone may have complicated our patient’s infection by exacerbating transient immunosuppression caused by EBV infection. Ceftriaxone therapy, in combination with surgical drainage, was effective in curing the brain abscesses, subdural empyema, and sinusitis. Arcanobacterium should be included in the differential diagnosis of brain abscesses in adolescents and young adults. Steroid therapy for uncomplicated EBV mononucleosis should be used with caution as transient immunosuppression may increase the risk for serious bacterial complications, including brain abscess.
